# A Community-Based Cross-Sectional Study on Awareness and Acceptance of Homosexuality in Coimbatore

**DOI:** 10.7759/cureus.59315

**Published:** 2024-04-29

**Authors:** Geetha Arumugam, Ramasamy Raja K, Divya B V, Nawin J Vignesh, Muthukumaran S

**Affiliations:** 1 Community Medicine, Karpagam Faculty of Medical Sciences and Research, Coimbatore, IND

**Keywords:** gender discrimination, social problem, general population, homophobia, lgbtq

## Abstract

Introduction: Acceptance of the lesbian, gay, bisexual, transgender, and queer/questioning (LGBTQ) community among the public varies greatly depending on cultural and social factors. The present study estimated homosexuality acceptance and the factors influencing its recognition among the general population in Coimbatore, Tamil Nadu, India.

Methods: This cross-sectional study was performed among people residing in urban and rural field practice areas of a tertiary care hospital in Coimbatore. Using a multistage random sampling method, people over 18 years of age were selected. A total of 670 individuals participated, and data was obtained. Data on sociodemographic characteristics and homosexuality acceptance were collected using the Homosexuality Attitude Scale.

Results: Overall, homosexuality acceptance was 61% among the community. Homosexuality acceptance was significantly associated with age (p<0.001), residence (p=0.014), marital status (p<0.001), religion (p<0.001), education (p=0.001), and occupation (p<0.001).

Conclusion: Overall acceptance was better among young participants as compared to the elders. Thus, our study highlights the need for a shift in perspective among the older generation, which may significantly improve their overall acceptance of homosexuality.

## Introduction

LGBTQ is an acronym that stands for lesbian, gay, bisexual, transgender, and queer/questioning [1]. This term represents various sexual orientations, gender identities, and expressions.

LGBTQ community members frequently encounter discrimination in various aspects of their lives [2]. Discrimination against the LGBTQ community can manifest in diverse forms, including legal, social, economic, and cultural discrimination.

Effects of such discrimination can have significant and far-reaching impacts on their well-being, mental health, and quality of life [3]. The impact of such discrimination can be both short term and long term, and it affects various aspects of an individual's personal and social life.

Discrimination may be associated with mental health issues such as depression and anxiety or physical health consequences such as substance abuse or risk for human immunodeficiency virus (HIV)/acquired immunodeficiency syndrome (AIDS). Family rejection and social exclusion can lower educational attainment and employment challenges. As a result, they develop stigma-driven secrecy upon attaining adulthood and indulge in hate crimes.

Some countries have made significant strides in addressing discrimination and protecting the rights of LGBTQ individuals [4]. India has had a conservative approach toward LGBTQ rights [5]. However, in 2018, the court made a landmark ruling where it struck down parts of Section 377, which decriminalized adult consensual same-sex relationships [6]. This ruling was a significant step toward recognizing the rights of the LGBTQ community in India.

Despite this legal victory, societal attitudes and acceptance of LGBTQ can vary across different regions and communities within India [7]. Many studies have identified the factors influencing the acceptance of homosexuality acceptance behavior [8-10]. Older males who are more religious and less educated show poor acceptance of LGBTQ individuals when compared with others [11].

Future studies and implementing antidiscrimination laws will help in identifying factors of discrimination. Following the decriminalization and protection of LGBTQ rights in India, there is no proper evidence of discrimination present against LGBTQ in the community. Therefore, this study would help to assess the existing discrimination level among the LGBTQ community following the decriminalization laws and identify the factors responsible for them.

## Materials and methods

An interview‑based cross‑sectional study was conducted among the general population residing in urban and rural areas of Coimbatore between September 2023 and January 2024. The data was collected from residents who were at least in Coimbatore for a year.

All participants were above the age of 18 years. Young people represent a country's future and are the main agents of change and progress. Therefore, preference was given to the youngest adult member of the family to participate in the study.

The sample size was calculated using a single proportion sample size formula. According to a study conducted by Lakshmi et al. [12], homosexuality acceptance among the general population was 59.3%. Considering a 95% confidence interval with a 4% margin of error, the sample size obtained was 580, and 670 individuals participated.

A multistage random sampling technique was used. The urban field practice area comprised three areas, and the rural field practice area comprised 17 villages. Probability proportional to size (PPS) sampling was used to select the number of households from each area and village. Later, every fifth house from each village/area was chosen using systematic random sampling. The interviewer visited the selected houses during non-working hours, preferably in the evening, to collect data. The study excluded houses that were locked on three consecutive visits. The contact details of the youngest member, who was unavailable during the visit to households, were obtained, and a telephonic interview was conducted after obtaining verbal consent.

Ethics committee approval and informed consent were obtained before the study's commencement. The first part of the questionnaire collected the study participants' demographic details. The second part contained the Homosexuality Attitude Scale [13]. It is a validated tool with Cronbach's alpha value of >.92 and has a good test-retest reliability (r=0.71). The Homosexuality Attitude Scale is a 21-item questionnaire using a five-point Likert scale to measure the level of homosexuality acceptance. The mean of all items was utilized to assess homosexuality acceptance with a maximum score of 5 (good acceptance) and a minimum score of 1 (poor acceptance). The scores were then categorized into good and poor acceptance, where a mean score of 3 and above was considered good acceptance, whereas below 3 was regarded as poor acceptance.

The data was entered in Microsoft Excel (Microsoft Corp., Redmond, WA) and analyzed using Statistical Package for the Social Sciences (SPSS) software version 26 (IBM SPSS Statistics, Armonk, NY). Descriptive statistics were represented in the form of frequencies and proportions. The association was tested using the chi-square test, and p<0.05 was considered statistically significant.

## Results

Of the 670 participants, 335 (50%) were collected from rural areas and 335 (50%) from urban areas. Approximately 272 (40.6%) were male, and 386 (57.6%) were female. The participants' mean age was 29.4 years, with a standard deviation of 11.9 years. Regarding marital status, 425 (63.4%) were unmarried or single, and 232 (34.6%) were married. In terms of religion, 86.3% of the study participants followed Hinduism, 9.3% followed Christianity, and 4.5% followed Islam.

In this study, 0.9% were illiterate, 1% were educated up to primary level (1st-5th grade), 5.4% were educated up to secondary education (6th-8th grade), 29.6% were educated until higher secondary or diploma, 46.3% were graduates, and 16.9% had received professional education.

Of the participants, 44.5% were students, 25.8% were professionals, 11.5% were semiprofessionals, and 6.3% were unemployed. According to the updated BG Prasad scale (2023), approximately 72.8% belonged to class 1, 17.9% belonged to class 2, and 4.6% belonged to class 3 socioeconomic status (Table 1).

**Table 1:** Sociodemographic characteristics of the study participants (N=670)

Characteristics	Frequency (number)	Percentage (%)
Age (years)	29.4 ± 11.9	
Gender		
Male	272	40.6
Female	386	57.6
Prefer not to mention	12	1.8
Residence		
Urban	335	50.0
Rural	335	50.0
Marital status		
Divorced/widowed/separated	13	1.9
Unmarried/single	425	63.4
Married	232	34.7
Religion		
Hindu	578	86.3
Christian	62	9.3
Muslim	30	4.5
Education		
Illiterate	6	0.9
Primary (1-5)	7	1.0
Secondary (6-10)	36	5.4
Higher secondary or diploma	198	29.6
Graduate	310	46.3
Professional	113	16.9
Occupation		
Student	298	44.5
Unemployed	42	6.3
Unskilled worker	17	2.5
Skilled worker	63	9.4
Semiprofessional	77	11.5
Professional	173	25.8
Socioeconomic status		
Class 1	488	72.8
Class 2	120	17.9
Class 3	31	4.6
Class 4	31	4.6

**Figure 1:**
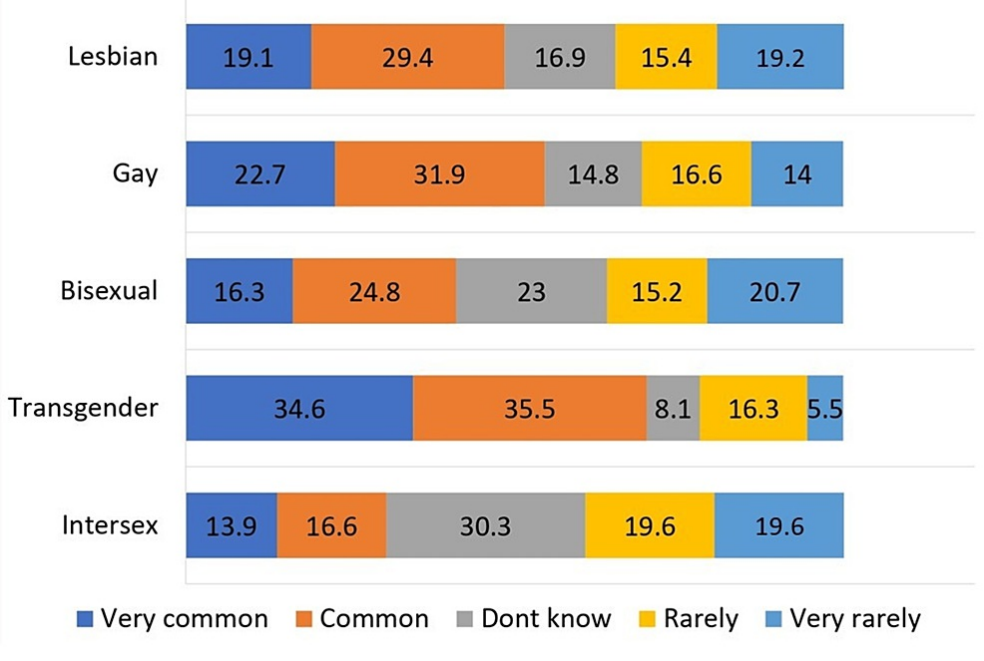
Levels of discrimination by the general population among various LGBTQ members (N=670) LGBTQ: lesbian, gay, bisexual, transgender, and queer/questioning

Figure 1 provides a comprehensive overview of perceived discrimination against the LGBTQ categories by the general population. Discrimination against lesbians was 48.5%, whereas against gay population was 54.6%. Bisexual individuals face discrimination at 41.1%, and the highest reported discrimination was for the transgender population with 70.1%. The discrimination against the queer population was 30.5%.

Table 2 shows the association between homosexuality acceptance and the study participants' sociodemographic characteristics. Homosexuality acceptance was good among females compared to males, with a p-value of 0.005. It was significantly associated with residence (p=0.014), marital status (p<0.001), religion (p<0.001), education (p=0.001), and occupation (p<0.001).

**Table 2:** Association of homosexuality acceptance with sociodemographic characteristics of the study participants (N=670)

Characteristics	Homosexuality acceptance		p-value
	Good acceptance (number (%))	Poor acceptance (number (%))	
Gender			0.005
Male	149 (54.8)	123 (45.2)	
Female	253 (65.5)	133 (34.5)	
Prefer not to mention	7 (58.3)	5 (41.7)	
Residence			0.014
Urban	213 (63.6)	122 (36.4)	
Rural	176 (52.6)	159 (47.4)	
Marital status			<0.001
Unmarried/single	287 (67.5)	138 (32.5)	
Married	117 (50.4)	115 (49.6)	
Religion			<0.001
Hindu	370 (64)	208 (36)	
Christian	29 (46.8)	33 (53.2)	
Muslim	10 (33.3)	20 (66.7)	
Education			0.001
Illiterate	2 (33.3)	4 (66.7)	
Primary (1-5)	1 (14.3)	6 (85.7)	
Secondary (6-10)	13 (36.1)	23 (63.9)	
Higher secondary or diploma	131 (66.2)	67 (33.8)	
Graduate	190 (61.3)	120 (38.7)	
Professional	72 (63.7)	41 (36.3)	
Occupation			<0.001
Student	207 (69.5)	91 (30.5)	
Unemployed	16 (38.1)	26 (61.9)	
Unskilled worker	7 (41.2)	10 (58.8)	
Skilled worker	26 (41.3)	37 (58.7)	
Semiprofessional	32 (41.6)	45 (58.4)	
Professional	121 (69.9)	52 (30.1)	
Socioeconomic status			0.114
Class 1	307 (62.9)	181 (37.1)	
Class 2	67 (55.8)	53 (44.2)	
Class 3	14 (45.2)	17 (54.8)	
Class 4	21 (67.7)	10 (32.3)	

**Figure 2:**
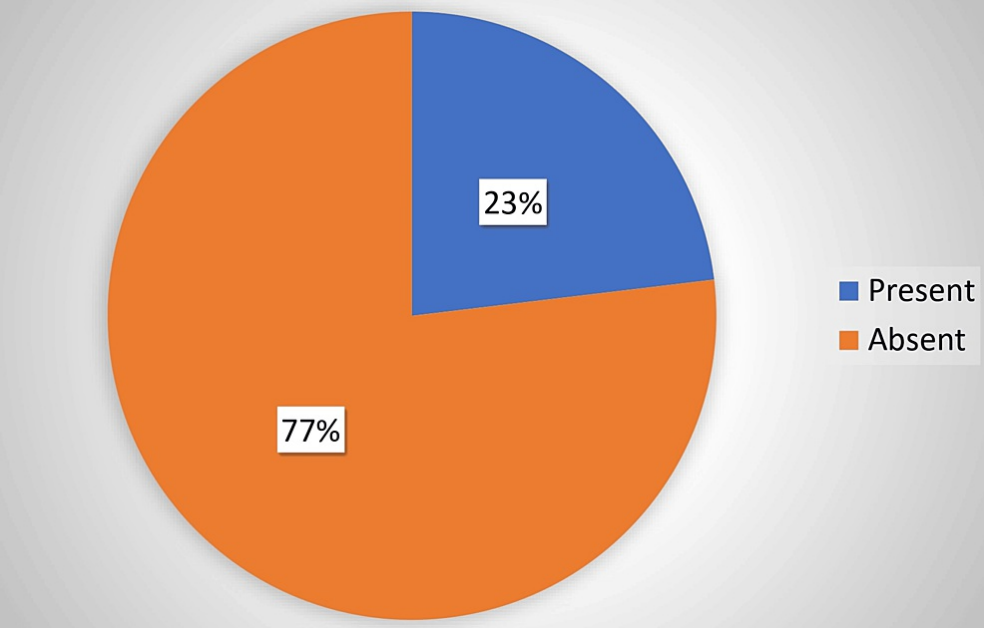
Distribution of participants' awareness of the national program for LGBTQ members (N=670) LGBTQ: lesbian, gay, bisexual, transgender, and queer/questioning

Figure 2 shows the participants' awareness of the national program for LGBTQ members. Although 23% of the participants were aware of the program, 77% of them were unaware of it.

**Figure 3:**
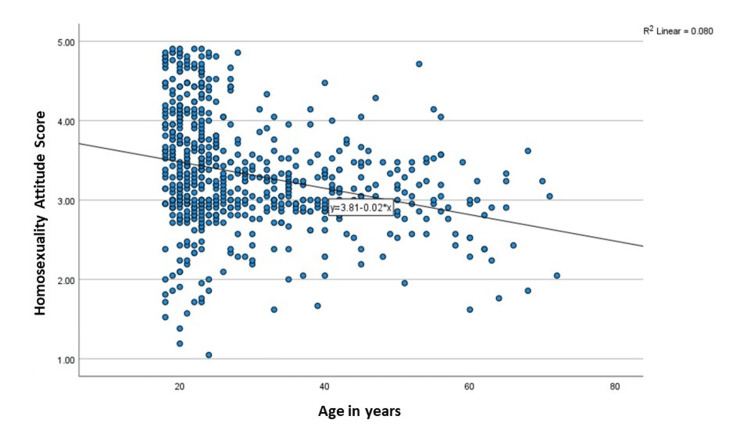
Scatter dot diagram showing acceptance of homosexuality with respect to age (N=670)

Figure 3 displays the scatter dot diagram with 670 data points and depicts the relationship between age (X-axis) (18-80 years) and homosexuality acceptance scores (Y-axis) (ranging from 1 to 5). The mean regression line has a slope of -0.02, implying a marginal decrease in acceptance (mean y=3.81 - 0.02x).

## Discussion

This study primarily aimed to estimate homosexuality acceptance among the general population and determine the influence of sociodemographic factors. The results confirmed our hypotheses and demonstrated that homosexuality acceptance is positively associated with the general population's sociodemographic characteristics.

Gender status has served as a crucial factor in homosexuality acceptance, which the current study continues to support. Overall, female respondents reported good homosexuality acceptance of 65.5%, while that of male respondents was 54.8%. This was proven in another study by West and Cowell [14], where female respondents reported less negative attitudes toward the LGBTQ community.

In our study, age was inversely correlated with homosexuality acceptance. This statement contradicts a study conducted among the general population in Jamaica, where age did not predict negative attitudes toward the LGBTQ community. The difference may be due to the influence of cultural variations [14].

Homosexuality acceptance provides significant information regarding the potential difference that exists between religions.

As individuals transition through different socioeconomic statuses, their income status changes. More than half of the individuals in all socioeconomic classes demonstrate good homosexuality acceptance, and no significant difference is evident among the classes. Although socioeconomic status does not show any significant difference, education and occupation have a significant association with homosexuality acceptance.

Some differences in homosexuality acceptance have been found between rural and urban residents, with rural residents reporting lower acceptance than rural residents. The reason may be that the rural population has a lower health literacy level than the urban population. According to a study by Banerjee [15], healthcare utilization among the rural population was poor compared to the urban population in India. Rural residents have lower access to health information, such as healthcare providers, magazines, and media.

Poor homosexuality acceptance can generate negative emotions among LGBTQ members, which significantly influence personal and social lives. Furthermore, it has a significant direct effect on the mental health of the LGBTQ population. Previous studies, such as those of Rubino et al. and Brewster et al., have demonstrated that education, pride, and social support are related to homophobic behavior. This evidence aligns with existing literature that promoting positive social relationships and community-based activities reduces homophobia and increases acceptance of the LGBTQ community [16,17].

Study limitations and future directions

Despite all the precautions, this study still has limitations. As the first limitation, this research followed a cross-sectional design that does not allow causal inferences on the relationships among the variables.

A second limitation concerns the additional variables that may influence the relationships in our framework, such as cultural factors. Despite these limitations, this research contributes to the existing literature. It highlights the influence of individuals' demographic characteristics on homophobic behavior.

Therefore, from a practical perspective, health education, support programs, and activities would help enhance homosexuality acceptance and reduce discrimination.

## Conclusions

Overall, homosexuality acceptance was 61% among the community. The overall acceptance was good among the young compared to the elders. Thus, the study highlights the need for a shift in perspective among the older generation, which may significantly improve overall acceptance. Future research should employ interventional trials to promote acceptance of the LGBTQ community.
